# Chlorotone as a Sedative in Gastric and Cystic Irritability

**Published:** 1901-03

**Authors:** C. F. Darnell

**Affiliations:** Llano, Texas


For Texas Medical Journal.

### Chlorotone as a Sedative in Gastric and Cystic Irritability.

BY. C. F. DARNELL, M. D., LLANO, TEXAS.

When I began to prescribe chloretone for my patients I did so tentatively, not expecting much in the way of results, because the drug was new and I was not familiar with its clinical effects. Among the first cases in which I had an opportunity to test its properties was one of alcoholism with insomnia, the chloretone having been given for the purpose of inducing sleep. The patient had been suffering with nausea and, as is usual in such cases, had vomited considerably. About the first effect I observed from the chloretone was its sedative influence upon the irritable stomach. In fact after giving a few doses the capability of the drug to control gastric irritability had been so clearly demonstrated that I resolved to make a further trial of it in other cases. The next occasion upon which I used chloretone was to check the vomiting in a case of uremic convulsions with anemia. Again the drug acted so well that I began to make use of it almost daily, and with entire satisfaction, in cases of nausea and vomiting from all manner of causes.

Although my experience in the use of chloretone as a local anesthetic has been limited, I can say that it has been perfectly satisfactory so far as it has extended. The most surprising result from its internal administration was met with in a case in which I had prescribed the new hypnotic to induce sleep. The patient suffered from nocturnal incontinence of urine and was compelled to rise frequently during the night to empty the bladder. Two ten-grain doses of chloretone, at night, so markedly relieved this symptom that I essayed to prescribe the drug in another case of a patient, well advanced in years, who had been afflicted sorely for a long time in a similar manner. The same dose was prescribed as in the previous case and with such a happy effect that the patient came back and wanted more of the medicine, remarking that it was just the thing he needed.

If a further investigation into this subject will show that chloretone is likely to prove useful in prostatic troubles and irritable bladders, that fact should be made known and utilized by the profession. I would rather recommend further clinical tests of chloretone by practitioners having hospital facilities, and I am satisfied that the published results will be useful to the entire profession. Of one thing I am certain and that is the peculiar value of chloretone in sub-acute gastritis from any cause.

